# Impact of Air-Drying Temperature on Antioxidant Properties and ACE-Inhibiting Activity of Fungal Fermented Lentil Flour

**DOI:** 10.3390/foods12050999

**Published:** 2023-02-27

**Authors:** Janaina Sánchez-García, Sara Muñoz-Pina, Jorge García-Hernández, Ana Heredia, Ana Andrés

**Affiliations:** 1 Instituto Universitario de Ingeniería de Alimentos para el Desarrollo (IUIAD-UPV), Universitat Politècnica de València, Camino de Vera s/n, 46022 Valencia, Spain; 2 Centro Avanzado de Microbiología de Alimentos (CAMA), Universitat Politècnica de València, Camino de Vera s/n, 46022 Valencia, Spain

**Keywords:** fermentation, *Pleurotus*, drying, flours, lentil, phenols, antinutrients

## Abstract

Solid-state fermentation (SSF) with *Pleurotus ostreatus* enhances the nutritional value of legumes. However, drying can cause significant changes in physical and nutritional properties of the final products. Thus, this work studies the impact of air-drying temperature (50, 60, and 70 °C) on relevant properties (antioxidant properties, ACE-inhibitory capacity, phytic acid, colour, and particle size) of two fermented lentils flour (Pardina and Castellana) using freeze-drying as a reference method. Castellana variety is a better substrate for *Pleurotus*, generating four times more biomass. In addition, an almost total reduction of phytic acid from 7.3 to 0.9 mg/g db is achieved in this variety. Air-drying significantly decreased the particle size and the final colour with ΔE > 20; nonetheless, the temperature does not play a crucial role. SSF decreased the total phenolic content and the antioxidant capacity regardless of the variety, however, drying at 70 °C increased total phenolic content (186%) in fermented Castellana flour. Comparing drying methods, freeze-drying implied a higher decrease in those parameters, reducing the TPC from 2.4 to 1.6 and from 7.7 to 3.4 mg gallic acid/g db in Pardina and Castellana dried flours. Finally, the flours inhibit the angiotensin I-converting-enzyme, and fermentation and drying increased their potential cardiovascular benefits.

## 1. Introduction

Legumes have been a cornerstone in Mediterranean cuisine and gastronomy; they are rich in proteins (usually 20%, reaching 30% in some varieties), carbohydrates (around 60%), vitamins, minerals, healthy fats (5%), and dietary fibres, but there has been a downward trend in their consumption [1]. Legume consumption has demonstrated a positive effect on health [2]. Furthermore, environmentally, legume cultivation promotes sustainable agriculture and contributes to climate change mitigation, and their ability to fix nitrogen can improve soil fertility and reduce the carbon footprint [3]. Moreover, they are also a more sustainable source of proteins (CO_2_ emissions of 1 kg of legume protein is 0.9 kg, while emissions of 1 kg of beef is 27 kg). However, its presence in the Mediterranean people’s diets has fallen to as low as 60–80 g/week, while the estimated recommendations are 200–250 g/week, according to the FAO. 

Solid-state fermentation has been recently explored as a strategy to preserve/enhance the nutritional value of legumes with promising results in obtaining new ingredients [4]. 

This kind of biotreatment has been recently applied in lentils, peas, soybeans, or quinoa, among others [5]. Up-to-date results show increased protein (18–23%) and antioxidant contents (30–53%) in the fermented flours, along with increased protein digestibility (12–17%) and a decrease in the carbohydrate content (6–29%). A novel approach to do so relates to the use of edible fungi as starter cultures (which has been usually applied for agro-residual materials valorisation) [6,7]. In a previous study, solid-state fermentation (SSF) with *Pleurotus ostreatus* proved to be an efficient way to enhance the nutritional profile of Pardina lentils and white quinoa [8]. Additionally, the potential health benefits due to the presence of fungal biomass support the bioconversion of legumes and pseudo-cereals by SSF. In fact, mushrooms have been proved to have good antioxidant capacities, and have demonstrated their inhibitory effects towards angiotensin I-converting enzyme (ACE) [9]. The use of natural antioxidants in the production of new foods is critical to maintaining adequate levels of antioxidants and ensuring balance regarding the prevention of pathologies. The inhibition of the ACE enzyme would reduce blood pressure, lowering the risk of hypertension complications [10]. This kind of bioprocessing could also be employed as a part of a multi-stage process, including stabilization unit operations (such as drying or milling, among others), to obtain promising flours from lentils. Lentil flours have been commonly used to nutritionally enhance food products as an ingredient used as a thickener, binder, gelling agent, and/or stabilizer [11,12]. Nowadays, there are different drying methods to obtain flour such as hot air recirculation, oven drying, tunnel drying, freeze-drying, spray drying, and different temperatures of drying. However, the drying process and temperature may have mixed influences on the final product due to the varied compositions and types of raw products. Drying processes can cause significant changes in the physical and nutritional properties of the final products [13,14]. Some drying methods might improve the quality, preservation, and value of raw materials, while others may provoke a significant decline [15]. Thus, the appropriate technology and conditions used for obtaining new dry products must be established separately for each type of food, based on the specific properties of the final product [16].

To this day, according to authors’ knowledge, there are no lentil fermented flours with *Pleurotus* in the bibliography, and no studies of the effect of drying on its physico-chemical properties. Thus, the aim of this work is to study the impact of fungal solid-fermentation and air-drying temperature (50, 60, and 70 °C) on the final properties of fermented lentils flours (Lens culinaris var. Pardina and Castellana) with *Pleurotus ostreatus*. Relevant properties such as antioxidant properties, ACE-inhibitory capacity, phytic acid, colour, and particle size have been analysed to provide recommendations for the usage of fermented lentil flours as prospective food ingredients.

## 2. Materials and Methods

### 2.1. Materials

Lentils (*Lens culinaris*) of Pardina and Castellana varieties (Hacendado^®^, Valencia, Spain were purchased at local stores in Valencia (Spain). The *Pleurotus ostreatus* strain was obtained from the Spanish Type Culture Collection (CECT20311). 

Phytic acid sodium salt hydrated from rice (C_6_H_18_O_24_P_6_·xNa^+^·yH_2_O), 2,2′-bipyridine (C_10_H_8_N_2_), thioglycolic acid (C_2_H_4_O_2_S,), sulphuric acid (H_2_SO_4_), sodium hydroxide (NaOH), sodium chloride (NaCl), 4-dimethylamino benzaldehyde (C_9_H_11_NO), acetylacetone (C_5_H_8_O_2_,), ascorbic acid (C_6_H_8_O_6_), ethyl acetate (C_4_H_8_O_2_), formic acid (CH_2_O_2_), 3,5-dinitrosalicylic acid (DNS) (C_7_H_4_N_2_O_7_), potassium sodium tartrate tetrahydrate (KNaC_4_H_4_O_6_·4H_2_O), folin ciocalteu reagent (C_6_H_6_O), 2,2′-azino-bis (3-ethylbenzothiazoline-6-sulphonic acid) (ABTS) (C_18_H_16_N_4_O_6_S_4_), 2,2-diphenyl-1-picrylhydrazyl (DPPH) (C_18_H_12_N_5_O_6_), 2,4,6-tripyridyl-s-triazine (TPTZ) (C_18_H_12_N_6_), (±)-6-Hydroxy-2,5,7,8-tetramethylchromane-2-carboxylic acid (Trolox) (C_14_H_18_O_4_), gallic acid (C_7_H_6_O_5_), Angiotensin Converting Enzyme (ACE) from rabbit lung (≥2.0 units/mg protein) (A6778-25UN), N-Hippuric-His-Leu hydrate (HHL), glucose (C_6_H_12_O6), mycopeptone, chloramphenicol, and tryptone bile X-glucuronide agar (TBX chromogenic selective medium) were obtained from Sigma-Aldrich Co. (St. Louis, MO, USA). For HPLC analysis, vanillic acid (C_8_H_8_O_4_), 4-hydroxybezoic acid (C_7_H_6_O_3_), rutin (C_27_H_30_O_16_), quercetin 3-glucoside (C_21_H_20_O_12_), quercitrin (C_21_H_20_O_11_), epicatechin (C_15_H_14_O_6_), quercetin (C_15_H_10_O_7_), trans-cinnamic acid (C_9_H_8_O_2_), naringenin (C_15_H_12_O_5_), 4-O-caffeoylquinic (C_16_H_18_O_9_), caffeic acid (C_9_H_8_O_4_), p-coumaric acid (C_9_H_8_O_3_), sinapic acid (C_11_H_12_O_5_), ferulic acid (C_10_H_10_O_4_), apigenin-7-glucoside (C_21_H_20_O_10_), and kaempferol (C_15_H_10_O_6_) were obtained also from Sigma-Aldrich Co. (St. Louis, MO, USA), all per analytical standards (HPLC grade). 

Ethanol absolute (C_2_H_6_O), concentrated hydrochloric acid (HCl), acetic acid glacial (C_2_H_4_O_2_), diethyl ether (C_2_H_5_OC_2_H_5_), ammonium iron (III) sulphate dodecahydrate (NH_4_Fe(SO_4_)_2_.12H_2_O), sodium carbonate (Na_2_CO_3_), and EDTA Calcium Disodium Salt (C_10_H_12_CaN_2_Na_2_O_8_) were obtained from Panreac AppliChem (Barcelona, Spain). Methanol (CH_4_O, HPLC grade), acetonitrile (C_2_H_3_N, HPLC grade), iron (III) chloride hexahydrate (FeCl_3_·6H_2_O), potassium persulphate (K_2_S_2_O_8_), and sodium acetate trihydrate (C_2_H_3_NaO_2_·3H_2_O) were obtained from Honeywell Fluka (Morris Plains, NJ, USA). Malt extract, agar, Plate-count agar, and Sabouraud dextrose agar were obtained from Scharlau (Barcelona, Spain).

### 2.2. Fungal Solid-State Fermentation

#### 2.2.1. Starter Culture Preparation

*Pleurotus ostreatus* mycelium from the stock culture was inoculated on malt agar petri dishes made with 2% malt extract, 2% glucose, 0.1% mycopeptone, and 1.5% agar, and then placed in an incubator (2001249, J.P. Selecta, Barcelona, Spain) at 28 °C for 14 days. The grown mycelium was inoculated in a culture broth made with 2% malt extract, 2% glucose, and 0.1% mycopeptone, and incubated at 28 °C for 14 days. A portion of the grown mycelium was taken and inoculated in a culture broth made with 2% malt extract, 2% glucose, and 0.1% mycopeptone, and incubated at 28 °C for 14 days.

For starter culture preparation, 10 g of Pardina and Castellana flour each was placed in petri dishes, hydrated to 65% moisture, and sterilised in an autoclave (4002136, J.P. Selecta, Barcelona, Spain) at 121 °C for 20 min. Finally, 1 mL of the culture broth containing the grown fungal mycelium was inoculated and incubated at 28 °C for 14 days.

#### 2.2.2. Fermentation Process

Solid-state fermentation was performed by placing 35 g of lentils (Pardina and Castellana) humidified to 65% moisture in glass jars and then sterilizing them at 121 °C for 20 min. The substrates were inoculated into the glass jars by adding 1/8 of the starter culture (petri dishes containing 10 g of colonised substrate divided into 8 portions) and then incubated at 28 °C for 14 days. Several glass jars were inoculated to obtain enough fermented substrate to perform all assays. Fermented substrates contained in glass jars were mixed to obtain a homogeneous sample for the subsequent drying process and analysis.

### 2.3. Drying and Milling of Fermented Grains/Seeds

Samples were dried by hot-air drying and lyophilization methods using a load of 500 g. Hot-air drying was performed using a convective dryer (Pol-Eko-Aparatura, CLW 750 TOP+, Kokoszycka, Poland) at three different drying temperatures (50, 60, and 70 °C), air velocity was 10.5 ± 0.2 m/s and air humidity percentage was 23.2 ± 2.9, 14.2 ± 1.7 and 8.7 ± 1.2 for 50, 60, and 70 °C, respectively. Lyophilization was conducted in a freeze dryer (Telstar, Lyoquest-55, Terrassa, Spain) at −45 °C and 0.8 mBar for 48 h. Milling was carried out with a food processor (Thermomix^®^, TM6-1, Wuppertal, Germany), applying 10,000 rpm at 15 s intervals for 1 min.

### 2.4. Drying Kinetics and Modelling

Drying kinetics was determined with a balance (Mettler Toledo, MS4002S, Greifensee, Zurich), by measuring the mass variation of the samples at each drying temperature (50, 60, and 70 °C) in a determined time interval, making several measurements over time until a constant weight was obtained. Drying curves of the samples were obtained, and an adjustment was made using the Lewis model equation (Equation (1)) for thin layer drying, which is one of the most common mathematical models used in the drying process of agricultural products [17,18].

(1) $$\frac{X_{t}^{w}}{X_{0}^{w}}=e^{-k\;\cdot \;t}$$

where: $X_{t}^{w}$ is the moisture at a determined time, $X_{0}^{w}$ is the moisture at time 0, *k* is the model constant, and *t* is the time (min).

### 2.5. Analytical Determinations

#### 2.5.1. Proximal Substrate Composition

Proximal composition of the substrate was conducted according to standardised methodologies of the Association of Official Analytical Chemists (AOAC) [19]. Moisture, protein, lipid, ash, and carbohydrate contents were analysed, the last one by difference. Total fibre, soluble, and insoluble fibre contents were determined according to the AOAC Method 991.43 and AACC Method 32-07.01. Results were expressed in g/100 g dry basis.

#### 2.5.2. Reducing Sugars

The methodology proposed by Miller [20] and Sansano et al. [21] for reducing sugars determination was used. An amount of 0.3 g of sample was mixed with 2 mL of 80% ethanol, vortexed, and allowed to stand for 30 min. It was centrifuged at 5000× *g* for 5 min (5810R, Eppendorf, Hamburg, Germany). The extraction was repeated twice, and the extracts were pooled. An aliquot of 500 µL was taken and mixed with 1 mL of DNS reagent (10 g/L of 3,5-dinitrosalicylic acid, containing 300 g potassium sodium tartrate tetrahydrate and 16 g sodium hydroxide). It was heated in a water bath (J.P.Selecta, Barcelona, Spain) at 100 °C for 5 min and then cooled to room temperature. The sample was diluted with 6 mL of distilled water and the absorbance was measured at 546 nm (Helios Zeta UV-VIS Spectrophotometer, Thermo Fisher Scientific, Waltham, MA, USA). Results were expressed as g glucose/100 g dry basis using a standard curve.

#### 2.5.3. Fungus Biomass

Biomass produced by the fungus was determined according to the method published by Aidoo et al. [22] and Tomaselli Scotti et al. [23]. Briefly, 100 mg of dry sample was mixed with 2.4 mL of 72% sulphuric acid at 25 °C for 24 h. Samples were diluted with 55 mL of distilled water and sterilised at 121 °C for 2 h. Then, the hydrolysate was neutralised to pH 7 with 10 M and 0.5 M sodium hydroxide with a pH meter (Mettler-Toledo, SevenCompact S210, Greifensee, Zurich). Erhlick’s reagent was prepared by dissolving 2.67 g of 4-dimethylamino benzaldehyde in 100 mL of a 1:1 mixture of ethanol reagent grade and concentrated hydrochloric acid. A 1 mL aliquot of previously neutralised hydrolysate was mixed with 1 mL of acetylacetone reagent (1 mL of acetylacetone and 50 mL of 0.5 M sodium carbonate) in a glass tube with a cap and heated in a boiling water bath for 20 min, then cooled to room temperature. Then, 6 mL of ethanol was added followed by 1 mL of Erhlick’s reagent. The mixture was incubated at 65 °C for 10 min, cooled to room temperature, and the absorbance was measured at 530 nm in a spectrophotometer (Beckman Coulter, DU 730, Brea, CA, USA). Results were expressed as mg glucosamine/g dry basis.

#### 2.5.4. pH and Water Activity (aw)

A 10% dilution of the samples was prepared and measured for pH determination. Water activity was measured with a dew point water activity meter (Decagon Devices Inc., Aqualab 4TE, Cervera, Spain) at 25 °C.

#### 2.5.5. Colour

Colour measurements of the different flours were carried out using a spectro-colourimeter (Minolta, CM-3600D, Tokyo, Japan), considering a standard illuminant D65 and a standard observer of 10°. The CIE-L*a*b*colour coordinates were measured. Tone (h) and chroma (C*) values were automatically calculated by the device with the a* and b* coordinates, and colour differences (ΔE) were calculated according to the following equation (Equation (2)):

(2) $$\Delta E=\sqrt{{\left(\Delta L^{*}\right)}^{2}+{\left(\Delta a^{*}\right)}^{2}+{\left(\Delta b^{*}\right)}^{2}}$$

#### 2.5.6. Particle Size

Particle size was measured with the dry method, using laser diffraction equipment (Mastersizer 2000, Malvern Instruments Limited, Malvern, UK). Results were reported as the equivalent volume mean diameter D[4.3] and percentile particle size d(0.5).

#### 2.5.7. Phytic Acid Content

Phytate content was determined using the method described by Haug and Lantzsch [24], and modified by Peng et al. [25]. A stock solution with 1.3 mg/mL phytic acid concentration was prepared and diluted with 0.2 M hydrochloric acid in the range of 0.1–1 mL (3.16–31.6 µg/mL phytate phosphorus). Ferric solution was prepared by dissolving 0.2 g of ammonium iron (III) sulphate dodecahydrate in 100 mL of 2 M hydrochloric acid and increased up to 1 L using distilled water. The 2,2′-bipyridine solution was prepared by dissolving 10 g of 2,2′-bipyridine and 10 mL of thioglycolic acid in distilled water and increased to 1 L. An amount of 50 mg of sample was mixed with 10 mL of 0.2 M hydrochloric acid and left overnight at 4 °C to prepare the extract. An amount of 1 mL of ferric solution and 500 µL of the extract were added in a tube. It was incubated in a boiling water bath for 30 min and then cooled to room temperature. The tube was centrifuged for 30 min at 3000× *g* and 1 mL of the supernatant was taken and mixed with 1.5 mL of 2,2′-bipyridine solution. The absorbance was measured at 519 nm against distilled water. A calibration curve was performed using a phytate reference solution. Assays were done in triplicate, and the results were expressed as mg phytic acid/g dry basis.

#### 2.5.8. Phenolic Compounds by HPLC Analysis

Phenolic compounds were extracted according to the methodology proposed by Caprioli et al. [26] and Giusti et al. [27]. To perform acid hydrolysis, 2.5 g of sample was weighed, and 7.5 mL of the extraction solvent (70:30 mixture of ethanol and bi-distilled water) was added. pH was adjusted with 2 M hydrochloric acid to pH 2 and subjected to an ultrasonic bath (J.P. Setecta, 3000840) for 2 h at room temperature. Samples were centrifuged at 8000× *g* for 15 min. The extraction was repeated twice. Both extracts obtained were pooled and filtered with a 0.45 µm PTFE filter, and subsequently, the free phenolic fraction was analysed by HPLC.

To conduct the alkaline hydrolysis, 14 mL of a mixture of 2 M sodium hydroxide with 0.01% 10 mM EDTA and 0.1% ascorbic acid was added to the acid hydrolysis residue (sediment) and left overnight to release bound phenolic esters or ethers. The pH was adjusted to 2 with 6 M hydrochloric acid and centrifuged at 8000× *g* for 15 min. Afterward, 15 mL of a mixture of ethyl acetate and diethyl ether in a 50:50 ratio was centrifuged at 5400× *g* for 10 min, and repeated twice. Both organic phases were pooled and concentrated in a rotary evaporator (Heidolph, Kelheim, Germany) at 25 °C. The concentrate was reconstituted with 10 mL of methanol, filtered with a 0.45 µm PTFE filter, and analysed by HPLC.

The obtained extracts were analysed using an HPLC 1200 Series Rapid Resolution coupled to a diode detector Serie (Agilent, Palo Alto, Santa Clara, CA, USA) following the methodology explained by Tanleque-Alberto et al. [28]. Phenolic compounds were separated on a Brisa-LC 5 µm C18 column (250 × 4.6 mm) (Teknokroma, Barcelona, Spain). Mobile phase A was 1% formic acid, and mobile phase B was acetonitrile (ACN). The following gradient program was established: 0 min, 90% A; 25 min, 40% A; 26 min, 20% A; holding until 30 min; 35 min, 90% A; holding until 40 min. The column working temperature, the flow rate and the injection volume were 30 °C, 0.5 mL/min, and 10 µL, respectively. Unknown compounds were identified by comparing the resulting chromatographic retention times with those of reference standards at the following wavelengths for each compound: 250 nm for vanillic acid; 260 nm for 4-hydroxibezoic acid, rutin, quercitin 3-glucoside, and quercitrin; 280 nm for gallic acid, epicatechin, quercetin, and trans-cinnamic acid; 290 nm for naringenin; 320 nm for 4-O-caffeoylquinic, caffeic acid, p-coumaric acid, sinapic acid, ferulic acid, and apigenin-7-glucoside; and 380 nm for kaempferol. Quantification of the identified compounds was carried out using a calibration curve by linear regression analysis of the area under the curve versus their concentration, and the results were calculated in µg/g dry weight.

#### 2.5.9. Total Phenolic Content

The total phenol content was determined with the Folin-Ciocalteu method described by Chang et al. [29], using the same extract described above for acid extraction in Section 2.5.8. An aliquot of 125 µL of the extract was taken, and 500 µL of bi-distilled water was added, followed by 125 µL of folin ciocalteu reagent, and left to react for 6 min. An amount of 1.25 mL of 7% sodium carbonate solution and 1 mL of bi-distilled water were added to reach a final volume of 3 mL. The mixture was incubated at room temperature in the dark for 30 min, and the absorbance was measured at 760 nm in a UV/Vis spectrophotometer. A standard curve for gallic acid was used, and the results were expressed as mg gallic acid/g dry basis.

#### 2.5.10. Antioxidant Activity

Antioxidant activity of the samples was determined by three different methods: ABTS, DPPH, and FRAP according to the methodology proposed by Thaipong et al. [30] with some modifications. The same acid extract described above was used.

For ABTS assay, stock solutions of 7.4 mM ABTS and 2.6 mM potassium persulphate were prepared, and then the working solution was prepared by mixing the two stock solutions in a 1:1 ratio and leaving them to react for 12 h at room temperature in darkness. After reaction, 1 mL of the fresh working solution is diluted with 60 mL of methanol to obtain an absorbance close to 1.1 at 734 nm in a UV/Vis spectrophotometer. An aliquot of 150 µL of the acidic extract was reacted with 2.85 mL of ABTS working solution for 2 h in the dark and the absorbance was measured at 734 nm.

For DPPH assay, a fresh working solution of 0.039 g/L DPPH in pure methanol was prepared to obtain an absorbance close to 1.1 at 515 nm in a UV/Vis spectrophotometer. An aliquot of 75 µL of the extract was reacted with 2.925 mL of DPPH working solution for 30 min in the dark and the absorbance was measured at 515 nm.

For FRAP assay, stock solutions of 300 mM acetate buffer (3.1 g sodium acetate trihydrate and 16 mL acetic acid glacial in 1 L water), pH 3.6, 10 mM TPTZ (2,4,6-tripyridyl-s-triazine) solution in 40 mM hydrochloric acid, and 20 mM iron (III) chloride hexahydrate solution were prepared. A fresh working solution was prepared by mixing acetate buffer, TPTZ solution, and iron (III) chloride hexahydrate solution in a 10:1:1 ratio, respectively, and incubated at 37 °C before use. An amount of 150 µL of the extract was reacted with 2.85 mL of FRAP working solution for 30 min in the dark, and the absorbance was measured at 593 nm.

In all three methods for antioxidant activity determination, a trolox standard curve was used, and the results were expressed as mg trolox/g dry basis. 

The overall antioxidant potency composite index (APCI) was determined by assigning all antioxidant activity assays (ABTS, DPPH, and FRAP) an equal weight by assigning an antioxidant index value of 100 to the highest sample score in each assay, and then calculating an antioxidant index for the other samples in each assay according to the following equation (Equation (3)) [31]:

(3) $$\mathrm{Antioxidant}\;\mathrm{index}\;\left(\%\right)=\left(\frac{\mathrm{sample}\;\mathrm{score}}{\mathrm{highest}\;\mathrm{sample}\;\mathrm{score}}\right)\times 100\;$$

Finally, the APCI was calculated by averaging each antioxidant activity assay’s antioxidant index (%) for each sample.

#### 2.5.11. Angiotensin-Converting Enzyme Inhibitory Activity (ACE ia (%))

ACE ia (%) of the samples was determined according to the method described by Akillioǧlu and Karakaya [32] and Hernández-Olivas et al. [33]. A double extraction of the protein was performed by mixing 5 g of the sample with 45 mL of distilled water, and the pH was adjusted to 11 and centrifuged at 10,000× *g* at 4 °C for 20 min. Supernatants were pooled, and the pH was adjusted to the isoelectric point (4.5), kept in gentle agitation for 1.5 h at 4 °C, and centrifuged at 10,000× *g* at 4 °C for 20 min. The sediment was dissolved in 50 mM phosphate buffer, pH = 7. Extracts were analysed immediately, otherwise, they were stored at −40 °C.

ACE reactive (25 mU/mL) and the substrate Hip-His-Leu (5 mM) were dissolved in 0.15 M Tris base buffer, containing 0.3 M sodium chloride, and the pH was adjusted at 8.3. Three controls (100 μL ACE + 40 μL distilled water; 140 μL distilled water; 40 μL sample extract +100 μL distilled water) were included together with the samples (100 μL ACE + 40 μL sample extract) and then incubated at 37 °C for 5 min. An amount of 100 μL substrate to each tube was added, and the incubation was continued for 30 min at the same temperature. An amount of 150 μL of 1 M hydrochloric acid was added to stop the reaction. An amount of 1 mL ethyl acetate was added and mixed vigorously in a vortex mixer. Samples were centrifuged 1200× *g* for 10 min, and 750 μL of the supernatant was collected and placed into clean tubes. Ethyl acetate contained in the supernatant was evaporated by gentle shaking at 80 °C. Solid hippuric acid contained in the tubes was dissolved in 1 mL of distilled water, and the absorbance was measured at 228 nm. ACE ia (%) was calculated according to the following equation (Equation (4)):

(4) ACE ia (%)=100−{100×(C−D)/(A−B)} 

where: A, B, C, and D are the absorbance of ACE + distilled water, distilled water, ACE + sample extract, and sample extract + distilled water, respectively.

#### 2.5.12. Microbiological Analysis

Samples of the different flours were collected aseptically to perform the corresponding microbiological analysis. For this, 1 g of each flour was diluted in 9 mL of sterile distilled water, and serial decimal dilutions were made. For the investigation of total aerobic mesophilic bacteria counts, mould, and yeast count, and Escherichia coli count, 0.1 mL of each serial dilution were plated onto Plate-count agar, Sabouraud dextrose agar with 50 mg/L of Chloramphenicol, and Tryptone Bile X-Glucuronide agar (TBX chromogenic selective medium), and incubated at 30 °C for 72 h, 25 °C for 5–7 days, and 44 °C for 24 h, respectively. Listeria monocytogenes and Salmonella spp. detection analyses were performed according to ISO 6579-1:2017 and ISO 11290-1:2017.

#### 2.5.13. Statistical Analysis

Experiments were carried out in triplicate and data were reported as mean ± standard deviation. One-way ANOVA and multiple range tests by the LSD procedure (least significant difference) of the Fisher test was performed to study possible differences between different drying temperatures using the statistical program Statgraphics Centurion version XV (Rockville, MD, USA) with a confidence level of 95% (*p*-value < 0.05).

## 3. Results and Discussion

### 3.1. Changes in Proximal Composition Induced by Fungal Solid-State Fermentation of Lentils

It is well known that legumes can be nutritionally modified by processing such as soaking, cooking, or dehydration, among others. Processed legumes show increases in protein digestibility, available starch, and soluble fibre, or important decreases in antinutritional factors such as phytic acid [34]. Solid-state fermentation processing implies a sequence of unit operations such as soaking and sterilising the substrate prior to the fermentation itself, so the nutritional properties of fermented legumes are expected to be different. Fungal solid-state fermentation of lentils with *Pleurotus ostreatus* provokes different changes depending on the characteristics of the initial substrates, mainly the lentil cultivar, which implies significant differences in composition, structure, and seed coat, to cotyledon ratio or seed size [5,35,36,37]. Table 1 shows the proximal composition of the substrates prior to and after the SSF process of both cultivars, as well as the biomass production, pH, and water activity. Since moisture content is the most affected component due to the conditioning process prior to inoculation and fermentation, the results are shown on a dry basis. Despite the slight differences between Pardina and Castellana cultivars in terms of initial composition, a clear difference in terms of biomass production was observed, being 14.1 and 61.8 mg of glucosamine/g dry basis, respectively, although the impact on the overall protein is not significant. These results point out that Castellana lentil is a better substrate for SSF with *Pleurotus ostreatus,* and the explanation could be found in the morphometric characteristics of these two cultivars. Plaza et al. [38] characterised the main Spanish lentil cult. They found that all of them, including the Castellana cultivar, showed a medium elliptic shape except for the cultivar Pardina which was classified as wide elliptic. The same authors reported the average weight (Pardina: 3.58 ± 0.24 and Castellana: 5.91 ± 0.15) and diameter (Pardina: 4.43 ± 0.09 and Castellana: 5.86 ± 0.09), which are morphometric properties affecting the density and porosity of the fermentation bed and the growing ability of mycelium.

*Pleurotus* is a lignocellulosic fungus, which means that it can depolymerize a complex structure made of cellulose, hemicelluloses, and lignin. That explains the reduction of total fibre observed in fermented samples of both cultivars (Table 1), especially the insoluble fraction. In addition, this process is also reflected in the increase in reducing sugars in both samples. In the case of the Castellana lentil, this increase is much higher, probably due to the greater amount of biomass present. The increase in the soluble fraction could be attributed to a solubilization process provoked by soaking and heating the substrates before inoculation. Aguilera et al. [34] observed similar results in Pardina lentils submitted to industrial dehydration processing, including previous soaking and cooking steps. The same authors observed lower ash values in processed flours than in raw lentils, coinciding with the ash reduction observed in fermented lentils, probably due to mineral losses during thermal processing in both cases. 

### 3.2. Air Drying Kinetics of Fermented Lentils

**Figure 1:**
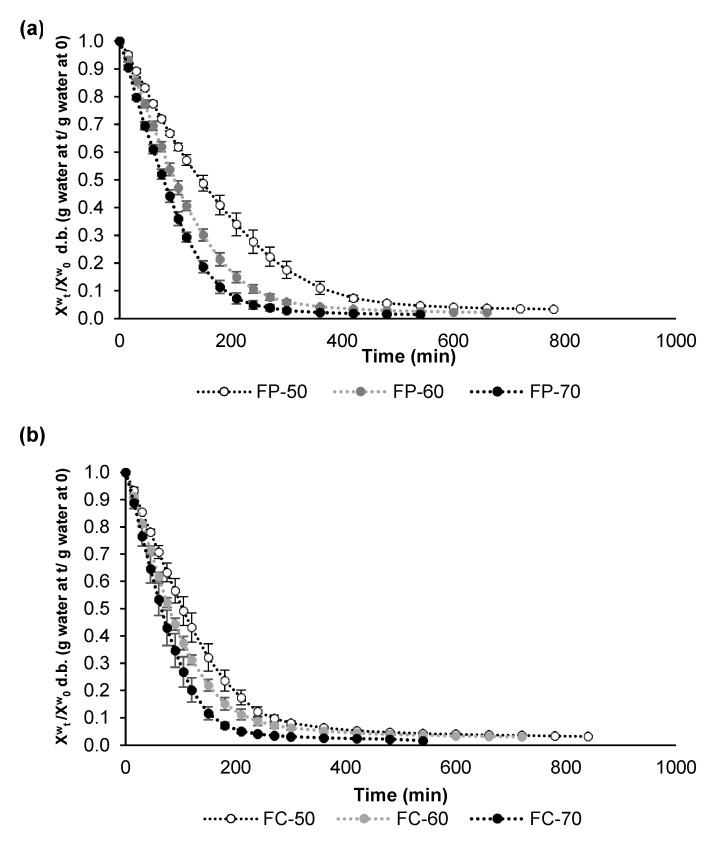
Air-drying curves (X^w^_t_/X^w^_0_ (g water at t/g water at 0)) at 50, 60 and 70 °C of: (**a**) fermented Pardina (FP), and (**b**) fermented Castellana (FC).

Fermented lentils were dried at different inlet air temperatures for modelling purposes. The drying curves obtained for both cultivars (Figure 1) revealed faster drying kinetics for Castellana lentils than Pardina. These results are in accordance with the results observed during soaking treatment before fermentation, in which water uptake was more rapid in Castellana lentils, revealing the higher moisture content of Castellana fermented lentils (Table 1) despite the same soaking time for both cultivars. Additionally, the impact of the air-drying temperature is higher in Pardina lentils. 

In the case of the Pardina lentil, drying with air at 50 °C demands approximately 9 h until the product reaches a constant weight. This time is reduced to 7 and 5.5 h when drying at 60 °C and 70 °C, respectively. In the case of the Castellana variety, the same tendency occurs, needing 8 and 6 h to get a constant weight after drying at the lowest temperatures. Increasing the temperature to 70 °C decreased the time required to 4.5 h. However, when obtaining a functional food ingredient, it is necessary to consider the effect of air-drying temperature on bioactive compound (phenols and antioxidants) content, processing time, and total energy consumption, which are key factors when setting up an industrial process. In this sense, drying at 70 °C would provide faster drying.

Air-drying curves were fitted following the Lewis model, and the constants and statistical coefficients of the model are shown in Table 2. In all cases, the R squared is superior to 0.97. The parameter K, related to the drying rate, shows a linear correlation with the air temperature. In the case of the Pardina lentil, the slope was 0.012, the intersection −0.348, and the coefficient of determination was 0.9974. On the other hand, for Castellana lentils, the slope was 0.010, the intersection −0.240, and the coefficient of determination was 0.9601.

These parameters were used to estimate the drying time needed at each temperature to obtain fermented lentil flours with 7% final moisture content. 

On the other hand, drying affects the microbiological quality of the product regardless of the air-drying properties. In fact, dehydration of the product reduces the amount of water available for microbial growth leading to microbial inhibition or even death. However, the temperature and speed of air drying can affect the rate of destruction of microbes. The microbiological quality parameters (Table 3) demonstrate that the obtained flours fit the minimal safety requirements. 

### 3.3. Impact of Processing on Particle Size, Colour, and Phytic Acid of Fermented Flours

**Figure 2:**
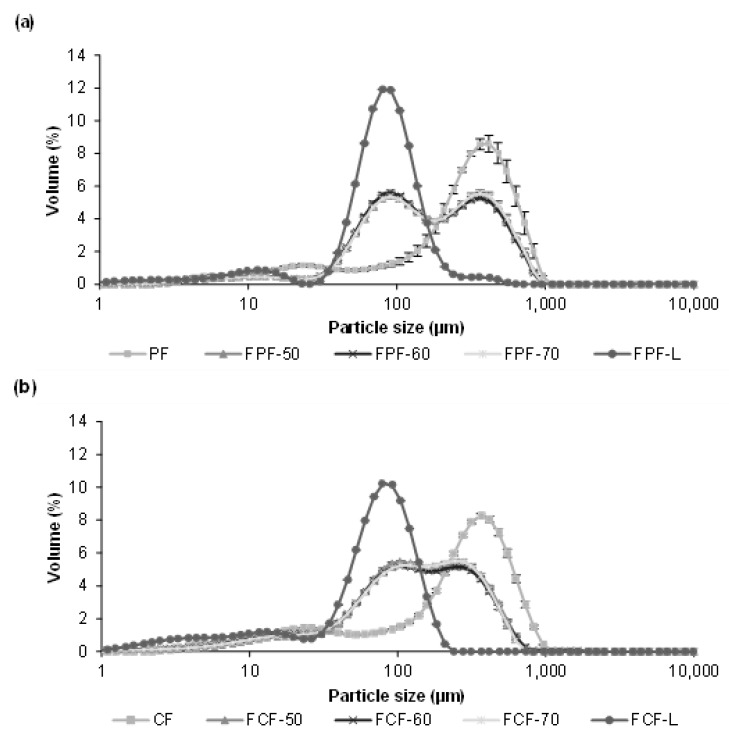
Particle size (volume (%)) in: (**a**) Pardina flour (PF) and fermented Pardina flour (FPF) obtained at different air-drying temperatures; (**b**) and Castellana flour (CF) and fermented Castellana flour (FCF) obtained at different air-drying temperatures. ^a,b,c,d^ Different lowercase letters indicate significant differences with a 95% (*p* < 0.05) significance level.

The drying conditions frequently affect the structure of dried foodstuffs [39], which is why the particle sizes of the resulting flours were affected by the drying process used during the previous dehydration step. Figure 2 shows the particle size distribution of fermented flours obtained by different drying processes for Pardina and Castellana cultivars.

Those flours obtained from fermented and dried lentils have a smaller particle size than raw lentil flours. Choe et al. [40] found the same results in thermally treated common bean flours, attributing this change to decreased seed hardness and less cohesiveness than the raw samples. In both cultivars, the same effect of fermentation and drying on the distribution and size of particles is observed. The drying method, air-drying or freeze-drying, significantly impacts the particle size, although the air-drying temperature does not affect this parameter. Raw lentil flour and fermented freeze-dried lentil flour present a monomodal distribution with an average particle size of 284 µm and 76.4 µm, respectively. Fermentation and hot-air drying shifted the particle size distributions towards smaller sizes, exhibiting a multimodal pattern. The reduction in particle size due to fermentation and drying was slightly more significant in the Castellana lentil flour. Nonetheless, Martini et al. [11] studied the influence of different particle sizes in red lentil flour for its use in bakery products. Although they found a slight influence on the water holding capacity—decreasing as the particle size is smaller—the multivariable statistics demonstrated that the particle size of the lentil flours is not the major factor affecting the rheology. In this case, technologically speaking, the reduction in particle size due to fermentation and drying would not be a problem.

One of the parameters in the assessment of flour colour is the L*a*b* difference (ΔE). This parameter significantly increased with the combined process of SSF and drying (Table 4). It can be observed that the substantial decrease in the L* value as well as the increase of the a* value compares to the unfermented flours in all cases. Changes in L* value and a* value could be related to Maillard reactions, caramelization, and/or pigment degradation [41] during the drying process. The drying method also significantly impacts the final colour of fermented lentil flours, with the freeze-dried fermented samples having fewer colour changes compared with the unprocessed one. 

The effect of SSF and drying on the final colour of flours was higher in Castellana variety than in Pardina, especially in the a* coordinate, which increases significantly. Similar results were found for dried apples [42], where the colour differences varied between 11.37 in the case of the freeze-dried apple and 21.11 in the case of air-drying at 60 °C. These differences in colour may be due to non-enzymatic Maillard reactions. Furthermore, the porous structure of dried products also differs, affecting mainly lightness of material due to the presence of air voids and pores [43].

### 3.4. Impact of Processing on Antioxidant and Anti- Hypertensive Properties of Fermented Flours

The potential health benefits of lentils have been attributed to secondary metabolites such as phenolic compounds, which exhibit antioxidant properties. These compounds can reduce the activity of reactive oxygen species by different mechanisms (scavenging the free radicals generated, complexing pro-oxidant metals, and quenching singlet oxygen) [34,44,45]. Table 5 shows the antioxidant activity evaluated by ABTS, DPPH, and FRAP, together with each antioxidant index, the APCI, and total phenol content. 

The results demonstrate significant differences between the phenolic compounds due to the processing (Table 5). It was found that processed Pardina samples were characterised by reduced phenolic content. At the same time, this parameter was higher for the hot-air dried Castellana variety samples without a significant effect on temperature. 

Comparing drying methods, freeze-drying implied a higher decrease in total phenol content than hot-air drying, regardless the variety. Similar results are found in literature when studying different legumes. In the case of bean sprouts, the total phenol content after being treated by hot-air drying was always significantly higher than that found when treated by freeze-drying either at low temperatures (20 °C) or high temperatures (80 °C) [46]. The same tendency was also found for pinto beans [47]. This reduction has been observed in other processes, such as boiling, and could be attributed to compound destruction, oxidation, or chemical rearrangement involving binding with other compounds [31]. Aguilera et al. [34] reported similar results under ordinary boiling for Pardina lentils. On the other hand, heat treatments were expected to increase TPC compounds due to the partial destruction of cellular structure and the subsequent release of bound compounds and/or the formation of Maillard reaction products with phenol and reducing agents above 40 °C [48]. Hot-air drying better retained phenolic compounds or underwent a higher release of those than freeze-drying in lentils. Regarding the antioxidant activity (ABTS, DPPH, and FRAP assays), similar trends were found exhibiting the relationship between these parameters and TPC (Table 5). Thus, fermentation and drying led to significant (*p* < 0.05) reduction in these activities with some exceptions. Only for Castellana lentils, an increase of FRAP and ABTS antioxidant activity was found in fermented flours due to hot-air drying. DPPH antioxidant activity, however, was the only parameter that rose because of fermentation for subsequently being negatively affected by drying. Que et al. 2008 [49] studied the influence of hot-air drying and freeze-drying on the antioxidant activities of pumpkin flours. They showed significantly higher total antioxidant activity in hot-air dried pumpkin flour than in freeze-dried flour. The authors attributed this to the creation of Maillard products or their intermediates with potent antioxidant activity. In our case, the initial content of reducing sugars is much higher in fermented Castellana than in fermented Pardina samples (see Table 1). This high concentration of reducing sugars can generate a greater number of compounds with greater antioxidant capacity after undergoing the Maillard reaction. Finally, among the processed flours, the highest APCI was obtained for fermented Castellana and Pardina flours air-dried at 70 °C with an APCI of 78.1 and 23.7%, respectively. 

Results of chromatogram profiles and concentrations of phenolic compounds identified, phenolic acids and flavonoids, are shown in Table 6 and Table 7 for Pardina and Castellana samples, respectively. In all cases, two extractions were performed to obtain phenolic compounds taking part in the free and bound fractions. However, in the case of Pardina samples, only unquantifiable traces were found for p-Coumaric acid, Epicatechin, and Ferulic acid for the second extraction (bound fraction). In the case of the Castellana lentils, Gallic acid, 4-Hydroxibezoic acid, and p-Coumaric acid as bonded phenols were detected. In this last case, both results were summed. Therefore, it can be said that most of the phenols are in their free form in the samples because of their native form, or due to a release along processing, fermentation and/or drying. This fact and the reduction in most phenols revealed that a destruction of these compounds is the main mechanism that occurred instead of rearrangement with other molecules or chemical conversion into other phenolic derivates in Pardina. The losses in Pardina samples are a consequence of the great reduction of 4-O-caffeoylquinic and Epicatechin, the most abundant phenolic acid and flavonoid, respectively, in raw Pardina lentils, through fermentation and further drying. Only a significant increase of vanillic acid and generation of trans-Cinnamic acid were found due to fermentation and hot-air drying. For Castellana samples, a notable increase in free gallic acid was seen during fermentation and drying, together with a drastic decrease in rutin and disappearing of epicatechin, among the most abundant compounds. As a result of these variations, a net increase of phenols was found in fermented hot-air dried samples, increasing as the drying temperatures increases.

It has been reported that some fungus genera, such as *Rhizopus* or *Aspergillus*, can synthesise microbial enzymes like tannases, and are able to release low-molecular weight phenolic acids from tanninic complex molecules [50,51]. This fact, together with the heating-induced depolymerization of condensed tannins, could also be responsible for the increase of some phenolic acids found in Pardina and Castellana fermented and dried samples.

On the other hand, legumes are rich in phytates, which have traditionally been considered a disadvantage since these compounds (present in legumes, whole grains, seeds, and nuts) hinder the absorption of specific vitamins and minerals (especially calcium, iron, and zinc). However, processing legumes are usually used to reduce antinutrients in legumes, and in this study, phytic acid content was analysed to assess the impact of SSF and drying temperature (Table 5). The initial content of phytic acid in the raw flours was within the range of the values published for these two cultivars [52], and in both cases, they were much lower than the harmful range of 10–60 mg/g [53].

The reduction in phytic acid can be attributed to hydrolysis by the endogenous phytase enzyme, which can be activated during processing. However, the reduction of these compounds because of processing (soaking, cooking, fermentation, drying, etc.) depends on the type of legume and the processing conditions [31]. In this study, significant differences between cultivars were observed in phytic acid reduction because of SSF. In contrast, the phytic acid content is almost negligible in fermented Castellana lentils; no significant differences were observed between raw and fermented Pardina lentils. This result reveals that the endogenous phytase enzyme in Pardina lentils is quickly inactivated by the autoclaving of the substrate before the inoculation and incubation for fermentation. The impact of drying on phytic acid content in the fermented flour was also analysed (Table 5). No significant differences can be attributed to the drying process, temperature, or drying type. The early inactivation of endogenous phytases in the Pardina during the processing before drying could explain these results, while Castellana’s endogenous phytase seems more resistant to processing inactivation. 

However, it should be noted that a moderate–small amount of phytates in the diet is even beneficial, since these compounds, in preclinical studies, have been shown to inhibit the proliferation of colon cancer cells [54]. Phytic acid, specifically hexaphosphate (inositol), is receiving special attention, because in cell and animal studies, it has been shown to have an anticancer action [55,56] in colon cancer, prostate cancer [57], and leukemia cells, among others [58]. Much remains to be investigated in this regard, but this shows that in cooked or germinated legumes, there is no problem with a certain amount of phytates, since it will hardly influence the absorption of nutrients and, on top of that, seems to bring us additional benefits.

**Figure 3:**
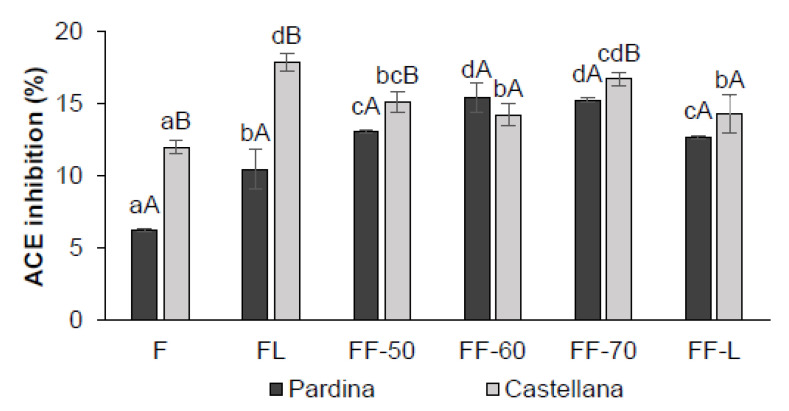
ACE inhibitory activity (%) values in Pardina and Castellana flour (F), fermented lentil (FL) and fermented flour (FF) dried by hot air (50, 60, and 70 °C), and lyophilization (L). Results represent the mean of three repetitions with their standard deviations. ^a,b,c,d^ Different lowercase letters indicate significant differences between dried flours and ^A,B^ capital letters indicate significant differences between lentil varieties with a significance level of 95% (*p* < 0.05).

Regarding the capacity of the samples to inhibit the angiotensin I-converting-enzyme (ACE), fermentation plus drying increased the potential cardiovascular benefits of the samples compared to their unprocessed lentils flours (Figure 3). 

The impact of processing was more notable on the Pardina than the Castellana variety; even though Castellana fermented and its dried flours exhibited a slightly, but statistically significant, higher ACE-inhibitory activity than Pardina samples. According to the literature, the ACE-inhibitory activity would correspond to protein fractions and mainly to low molecular weight peptides. Some plant proteins have been reported to be a source of various bioactive ACE-inhibitory peptides with anti-hypertensive activity. This is the case for some protein hydrolysates or isolates from soybean, bean, pea, sesame, rice and zein [59]. Moreover, increased dietary intake of plant protein was reported to exert a more beneficial effect on blood pressure compared to protein from animals [59]. It is also noticeable how only the fermentation with *Pleurotus* significantly increases the ACE-inhibitory capacity in both varieties of lentils by about five percentage points. *Pleurotus ostreatus* is well known for its potential in ACE inhibition. Abdullah et al. [9] reported that all *Pleurotus* fungi studied performed ACE inhibition, mainly due to its protein content. The effect of the type of drying on ACE capacity also performs an influence. In our case, the lyophilization performs lesser activity than the lentils dried at 70 °C. Piskov et al. [10] studied various drying methods on the ACE inhibition activity of *Pleurotus Ostreatus* and concluded that *P.Ostreatus* dried using freeze-drying exhibited a lower ACE inhibitory capacity than the same mushroom dried with hot air in agreement with our data.

On the other hand, Mohamad Ansor et al. [60] reported the capability of some mycelia such as of *G. lucidum* in lowering blood pressure levels. Apparently, four proteins (cystathionine beta synthase-like protein, DEAD/DEAH box helicase-like protein, paxillin-like protein, and alpha/beta hydrolase-like protein) derived from edible mushrooms would be responsible for the ACE inhibition. Therefore, the partial hydrolysis of native proteins along fermentation together with the mycelium could be responsible for an improvement in the benefits to cardiovascular health of the samples. Several studies have established a relationship between the chemical structure of peptides and their ability to inhibit ACE [61]. Peptides with hydrophobic or aromatic terminal amino acids are more likely to interact with the active site of ACE and present highest ACE inhibitory activity. The presence of C-terminal aromatic amino acid residues and N-terminal hydrophobic amino acid residues can also enhance the peptide’s activity in inhibiting ACE [61].

## 4. Conclusions

In the present study, the implications of solid-state fermentation (SSF) together with stabilization (by hot-air or freeze-drying) on some functional and technological properties, which play a relevant role in ingredients’ quality, were evaluated in fermented lentil flours. The obtained results demonstrated the Castellana variety was the most suitable substrate for fungal solid-state fermentation using edible fungus *Pleurotus ostreatus*, with a significant reduction in antinutrient phytic acid (from 7.3 to 0.9 mg/g db) as well as insoluble fibre (15 to 11 g/100 g db). Additionally, the SSF increased the ACE inhibitory capacity in both varieties of lentils by about five percentage points. With respect to dehydration and hot-air drying, specifically at 70 °C, there was an increase in total phenolic content, inhibition of ACE, and lower phytic acid content in fermented Castellana lentils compared to freeze-drying. Drying significantly decreased the particle size from around 270 µm to less than 150 µm, reaching only 76.4 µm after lyophilization. Fermented freeze-dried lentil flour presents a monomodal distribution in both varieties of lentil, while air-drying flours shifted the particle size distributions towards smaller sizes, exhibiting a multimodal pattern. Colour changes have also been notable after fermentation and drying (ΔE > 20), being more pronounced when lyophilizing. Regarding the phenolic profile, it has been observed how fermentation changes the profile, decreasing some compounds and increasing others, such as p-coumaric acid, vanillic acid, and quercetin for the Pardina variety and gallic acid, trans-cinnamic acid, and naringenin for the Castellana variety. Regarding the antioxidant capacity, the SSF negatively affected the antioxidant capacity of lentils, however, air-drying processing at 70 °C significantly increased the values obtained by ABTS and FRAP in the case of Castellana lentil flour. Therefore, fermented Castellana flours obtained by solid-state fermentation with *P.ostreatus* and air-dried at 70 °C might be considered a promising rich protein ingredient with improved functionality and new optical properties. In vitro digestion evaluation, however, could be important to go deeper into the healthy benefits of these flours. 

## Figures and Tables

**Table 1:** Proximate composition (g/100 g dry basis) in Pardina (P) and fermented Pardina (FP), and Castellana (C) and fermented Castellana (FC).

	Pardina (P)	Fermented Pardina (FP)	Castellana (C)	Fermented Castellana (FC)
Moisture	6.78 ± 0.06 ^a^	118.25 ± 0.17 ^b^	8.66 ± 0.05 ^a^	156.4 ± 0.3 ^b^
Protein	23.78 ± 0.4 ^a^	24.1 ± 0.6 ^a^	26.4 ± 0.2 ^a^	26.9 ± 0.4 ^a^
Lipids	1.06 ± 0.09 ^a^	1.31 ± 0.11 ^b^	1.38 ± 0.06 ^a^	1.68 ± 0.13 ^b^
Ashes	2.74 ± 0.02 ^b^	2.481 ± 0.014 ^a^	3.35 ± 0.02 ^b^	2.596 ± 0.009 ^a^
Total Carbohydrates *	72.32 ± 0.4 ^a^	72.1 ± 0.7 ^a^	68.9 ± 0.2 ^a^	68.9 ± 0.5 ^a^
Reducing sugars	0.22 ± 0.01 ^a^	0.92 ± 0.08 ^b^	0.22 ± 0.02 ^a^	2.94 ± 0.18 ^b^
Total fibre	16.0 ± 0.2 ^b^	14.3 ± 0.2 ^a^	17.3 ± 0.2 ^b^	13.6 ± 0.3 ^a^
Soluble fibre	1.39 ± 0.10 ^a^	2.25 ± 0.10 ^b^	1.94 ± 0.10 ^a^	1.78 ± 0.10 ^a^
Insoluble fibre	15.0 ± 0.3 ^b^	12.26 ± 0.10 ^a^	15.1 ± 0.2 ^b^	11.5 ± 0.2 ^a^
Biomass **	-	14.1 ± 1.5	-	61.8 ± 1.4
pH	6.457 ± 0.006 ^a^	7.217 ± 0.006 ^b^	6.603 ± 0.006 ^b^	6.520 ± 0.010 ^a^
a_w_	0.367 ± 0.003 ^a^	0.9798 ± 0.0011 ^b^	0.5084 ± 0.0012 ^a^	0.9736 ± 0.0008 ^b^

Results represent the mean of three repetitions with their standard deviation. ^a,b^ Different lowercase letters indicate significant differences with a 95% (*p* < 0.05) significance level. * Carbohydrates calculated by difference; ** (mg glucosamine/g dry basis).

**Table 2:** Drying kinetic parameters k and R squared at different temperatures.

Temperature (°C)	Fermented Pardina		Fermented Castellana	
	k	R^2^	k	R^2^
50	0.2218	0.9788	0.2854	0.9746
60	0.3467	0.9771	0.3519	0.9768
70	0.4514	0.9698	0.4910	0.9732

**Table 3:** Microbiological analysis in Pardina flour (PF) and Castellana flour (CF), and fermented Pardina flour (FPF) and fermented Castellana flour (FCF) dried by hot air (50, 60 and 70 °C) and lyophilization (L).

	Aerobic Mesophilic Bacteria count (UFC/g)	Yeast and Mould Count (UFC/g)	*E. coli* Count (UFC/g)	*Salmonella* spp. Detection (Presence /Absence)	*Listeria monocytogenes* (Presence /Absence)
PF	2.5 × 10^3^	˂10^2^	˂10^2^	Absence	Absence
FPF-50	5 × 10^2^	1 × 10^2^	˂10^2^	Absence	Absence
FPF-60	˂10^2^	˂10^2^	˂10^2^	Absence	Absence
FPF-70	˂10^2^	˂10^2^	˂10^2^	Absence	Absence
FPF-L	3 × 10^2^	˂10^2^	˂10^2^	Absence	Absence
CF	6 × 10^2^	˂10^2^	˂10^2^	Absence	Absence
FCF-50	4 × 10^2^	˂10^2^	˂10^2^	Absence	Absence
FCF-60	1 × 10^2^	˂10^2^	˂10^2^	Absence	Absence
FCF-70	˂10^2^	˂10^2^	˂10^2^	Absence	Absence
FCF-L	4 × 10^2^	˂10^2^	˂10^2^	Absence	Absence

**Table 4:** Colour of obtained flours from Pardina flour (PF) and Castellana flour (CF), fermented Pardina (FP) and fermented Castellana (FC), and fermented Pardina flour (FPF) and fermented Castellana flour (FCF) dried by hot air (50, 60 and 70 °C), and lyophilization (L).

Pardina Flours			Castellana Flours		
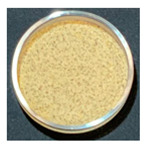 **PF**	L*	80.2 ± 0.3 ^d^	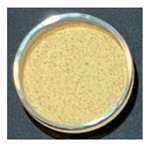 **CF**	L*	82.86 ± 0.09 ^d^
	a*	1.70 ± 0.06 ^a^		a*	0.905 ± 0.007 ^a^
	b*	15.5 ± 0.2 ^a^		b*	18.34 ± 0.10 ^e^
	C*	15.5 ± 0.2 ^d^		C*	18.36 ± 0.10 ^e^
	h	83.72 ± 0.15 ^d^		h	87.175 ± 0.008 ^c^
	ΔE	-		ΔE	-
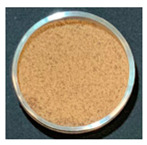 **FPF-50**	L*	61.9 ± 0.2 ^b^	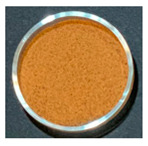 **FCF-50**	L*	58.7 ± 0.4 ^a^
	a*	4.42 ± 0.03 ^c^		a*	7.18 ± 0.11 ^d^
	b*	11.22 ± 0.09 ^ab^		b*	15.63 ± 0.09 ^b^
	C*	12.06 ± 0.10 ^ab^		C*	17.20 ± 0.13 ^b^
	h	68.49 ± 0.04 ^b^		h	65.3 ± 0.2 ^a^
	ΔE	18.96 ± 0.41 ^b^		ΔE	25.1 ± 0.4 ^c^
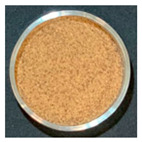 **FPF-60**	L*	61.73 ± 0.06 ^ab^	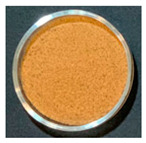 **FCF-60**	L*	60.4 ± 0.2 ^b^
	a*	4.30 ± 0.02 ^b^		a*	6.972 ± 0.012 ^c^
	b*	10.91 ± 0.05 ^a^		b*	16.18 ± 0.08 ^c^
	C*	11.73 ± 0.06 ^a^		C*	17.61 ± 0.07 ^c^
	h	68.48 ± 0.03 ^b^		h	66.68 ± 0.14 ^b^
	ΔE	19.20 ± 0.23 ^b^		ΔE	23.4 ± 0.2 ^b^
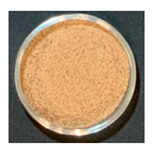 **FPF-70**	L*	61.3 ± 0.3 ^a^	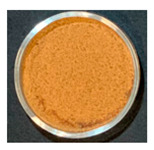 **FCF-70**	L*	58.37 ± 0.14 ^a^
	a*	4.43 ± 0.07 ^c^		a*	7.44 ± 0.02 ^e^
	b*	11.44 ± 0.19 ^b^		b*	16.38 ± 0.09 ^d^
	C*	12.3 ± 0.2 ^b^		C*	17.99 ± 0.07 ^d^
	h	68.83 ± 0.02 ^c^		h	65.57 ± 0.17 ^a^
	ΔE	19.51 ± 0.46 ^b^		ΔE	25.4 ± 0.2 ^c^
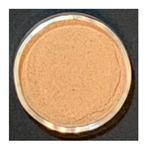 **FPF-L**	L*	62.5 ± 0.3 ^c^	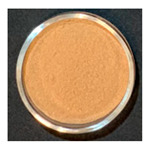 **FCF-L**	L*	63.01 ± 0.04 ^c^
	a*	5.01 ± 0.09 ^d^		a*	6.51 ± 0.02 ^b^
	b*	12.6 ± 0.3 ^c^		b*	14.32 ± 0.03 ^a^
	C*	13.6 ± 0.3 ^c^		C*	15.73 ± 0.03 ^a^
	h	68.30 ± 0.09 ^a^		h	65.55 ± 0.06 ^a^
	ΔE	18.23 ± 0.17 ^a^		ΔE	21.02 ± 0.03 ^a^

Results represent the mean of three repetitions with their standard deviation. ^a,b,c,d,e^ Different lowercase letters indicate significant differences for each parameters L*, a*, b*, C*, h, and ΔE for Pardina and Castellana with a 95% (*p* < 0.05) significance level.

**Table 5:** Antioxidant activity (mg trolox/g dry basis) by ABTS, DPPH, and FRAP method, total phenol content (mg gallic acid/g dry basis), and phytic acid content (mg/g dry basis) in Pardina flour (PF) and Castellana flour (CF), fermented Pardina (FP) and fermented Castellana (FC), and fermented Pardina flour (FPF) and fermented Castellana flour (FCF) dried by hot air (50, 60 and 70 °C) and lyophilization (L).

	Antioxidant Activity							Total Phenol Content	Phytic Acid
	ABTS	ABTS Index	DPPH	DPPH Index	FRAP	FRAP Index	APCI *		
PF	9.5 ± 0.4 ^d^	100	2.07 ± 0.09 ^c^	100	7.62 ± 0.17 ^b^	100	100	3.8 ± 0.2 ^c^	9.7 ± 0.7 ^b^
FP	5.7 ± 0.5 ^c^	60.7	0.64 ± 0.04 ^b^	30.8	0.311 ± 0.019 ^a^	4.09	31.9	2.10 ± 0.08 ^b^	7.3 ± 0.6 ^a^
FPF-50	3.81 ± 0.10 ^b^	40.3	0.49 ± 0.03 ^a^	23.6	0.30 ± 0.02 ^a^	3.9	22.6	2.21 ± 0.09 ^b^	7.4 ± 1.4 ^a^
FPF-60	4.03 ± 0.19 ^b^	42.7	0.486 ± 0.016 ^a^	23.5	0.32 ± 0.02 ^a^	4.2	23.5	2.39 ± 0.19 ^b^	7.6 ± 1.2 ^a^
FPF-70	3.91 ± 0.16 ^b^	41.4	0.516 ± 0.010 ^a^	25	0.351 ± 0.007 ^a^	4.6	23.7	2.37 ± 0.12 ^b^	7.7 ± 0.9 ^a^
FPF-L	3.20 ± 0.04 ^a^	33.9	0.502 ± 0.014 ^a^	24.3	0.310 ± 0.16 ^a^	4.07	20.7	1.59 ± 0.08 ^a^	8.0 ± 1.4 ^ab^
CF	8.4 ± 0.4 ^d^	100	1.634 ± 0.015 ^bc^	72	8.3 ± 0.2 ^e^	100	90.7	4.13 ± 0.10 ^c^	7.3 ± 0.3 ^d^
FC	2.50 ± 0.09 ^a^	29.9	2.27 ± 0.13 ^d^	100	1.10 ± 0.03 ^a^	13.4	47.7	2.75 ± 0.11 ^a^	0.9 ± 0.5 ^c^
FCF-50	6.1 ± 0.3 ^c^	73.2	1.61 ± 0.02 ^b^	70.8	6.21 ± 0.19 ^c^	75.2	73.1	6.9 ± 0.3 ^d^	0.4 ± 0.4 ^bc^
FCF-60	5.48 ± 0.07 ^b^	65.4	1.568 ± 0.016 ^b^	69.07	6.3 ± 0.2 ^c^	76.2	70.2	7.13 ± 0.12 ^e^	0.21 ± 0.07 ^b^
FCF-70	6.2 ± 0.2 ^c^	73.9	1.71 ± 0.02 ^c^	75.1	7.0 ± 0.3 ^d^	85.3	78.1	7.71 ± 0.15 ^f^	0.02 ± 0.01 ^a^
FCF-L	2.32 ± 0.16 ^a^	27.7	1.093 ± 0.016 ^a^	48.2	2.14 ± 0.05 ^b^	25.9	33.9	3.42 ± 0.02 ^b^	0.26 ± 0.04 ^b^

Results represent the mean of three repetitions with their standard deviation. ^a,b,c,d,e,f^ Different lowercase letters indicate significant differences with a 95% (*p* < 0.05) significance level. *APCI: Antioxidant potency composite index.

**Table 6:** Phenolic content (µg/g dry weight) in raw Pardina flour (PF) and fermented Pardina (FC), dried at 50, 60, and 70 °C (FPF-50, FPF-60, FPF-70), and lyophilised (FPF-L).

	PF	FP	FPF-50	FPF-60	FPF-70	FPF-L
Phenolic acids						
Gallic acid	n.d.	n.d.	n.d.	n.d.	n.d.	n.d.
Caffeic acid	10.6 ± 0.4 ^BC^	n.d.	n.d.	n.d.	n.d.	n.d.
p-Coumaric acid	7.8 ± 0.2 ^bABC^	9.47 ± 0.04 ^dC^	8.5 ± 0.5 ^cC^	10.2 ± 0.5 ^dC^	9.5 ± 0.2 ^dC^	5.2 ± 0.1 ^aA^
Sinapic acid	n.d.	n.d.	n.d.	n.d.	n.d.	n.d.
4-O-Caffeoylquinic	264 ± 6 ^dG^	143.5 ± 0.06 ^cF^	121 ± 3 ^aG^	123 ± 2 ^aF^	127 ± 3 ^bF^	152 ± 5 ^cD^
4-Hydroxibezoic acid	11.0 ± 0.2 ^aC^	11.78 ± 0.03 ^aD^	11.0 ± 0.3 ^aD^	11.2 ± 0.3 ^aC^	10.7 ± 0.3 ^aC^	10.1 ± 0.1 ^aB^
Vanillic acid	23.0 ± 0.3 ^aE^	34.7 ± 0.9 ^bE^	47 ± 3 ^cF^	47 ± 2 ^cE^	46 ± 4 ^cE^	34 ± 2 ^bC^
Ferulic acid	6.6 ± 0.2 ^cA^	5.3 ± 0.1 ^bA^	2.8 ± 0.01 ^aA^	2.92 ± 0.03 ^aA^	2.88 ± 0.06 ^aA^	2.90 ± 0.03 ^aA^
trans-Cinnamic acid	traces	6.6 ± 0.2 ^bB^	20.78 ± 0.04 ^cE^	29 ± 2 ^dD^	26.8 ± 0.6 ^dD^	4.98 ± 0.05 ^aA^
Flavonoids						
Rutin	11.6 ± 0.8 ^C^	n.d.	n.d.	n.d.	n.d.	n.d.
Epicatechin	71 ± 2 ^F^	n.d.	n.d.	n.d.	n.d.	n.d.
Quercetin 3-glucoside	n.d.	n.d.	n.d.	n.d.	n.d.	n.d.
Quercitrin	6.70 ± 0.02 ^cAB^	traces	3.38 ± 0.05 ^aAB^	3.30 ± 0.03 ^aA^	3.2 ± 0.2 ^aA^	3.76± 0.09 ^bA^
Apigenin-7-glucoside	16.9 ± 0.1 ^D^	traces	traces	traces	traces	Traces
Quercetin	5.7 ± 0.2 ^cA^	11.2 ± 0.1 ^dD^	5.89 ± 0.07 ^aB^	6.05 ± 0.03 ^aB^	5.95 ± 0.03 ^aB^	5.76 ± 0.05 ^bA^
Naringenin	n.d.	n.d.	n.d.	n.d.	n.d.	n.d.
Kaempferol	n.d.	n.d.	n.d.	n.d.	n.d.	n.d.

^a,b,c,d^ Different lowercase letters indicate significant differences (*p* < 0.05) between flours. ^A,B,C,D,E,F,G^ Different capital letters indicate significant differences (*p* < 0.05) between phenolic compounds. n.d.: not detected. Traces: not quantifiable.

**Table 7:** Phenolic content (µg/g dry weight) in raw Castellana flour (CF) and fermented Castellana (FC), dried at 50, 60, and 70 °C (FCF-50, FCF-60, FCF-70), and lyophilised (FCF-L).

	CF	FC	FCF-50	FCF-60	FCF-70	FCF-L
Phenolic acids						
Gallic acid	45 ± 5 ^aE^	67 ± 2 ^bD^	143 ± 10 ^dE^	129 ± 15 ^dE^	181.6 ± 0.8 ^eE^	102 ± 7 ^cE^
Caffeic acid	13 ± 2 ^bC^	8.2 ± 0.5 ^aC^	8 ± 0.2 ^aC^	7.6 ± 0.4 ^aC^	8.1 ± 0.8 ^aBC^	8.3 ± 0.5 ^aC^
p-Coumaric acid	9 ± 0.1 ^C^	traces	traces	traces	traces	Traces
Sinapic acid	n.d.	n.d.	n.d.	n.d.	n.d.	n.d.
4-O-Caffeoylquinic	n.d.	n.d.	n.d.	n.d.	n.d.	n.d.
4-Hydroxibezoic acid	4.5 ± 0.2 ^aB^	9.8 ± 0.4 ^bC^	12.1 ± 0.6 ^cD^	12.2 ± 0.3 ^cD^	10 ± 1 ^bC^	12.5 ± 0.5 ^cD^
Vanillic acid	2.7 ± 0.2 ^A^	n.d.	n.d.	n.d.	n.d.	n.d.
Ferulic acid	n.d.	n.d.	n.d.	n.d.	n.d.	n.d.
trans-Cinnamic acid	n.d.	5.9 ± 0.7 ^aB^	9.7 ± 0.2 ^bCD^	11.5 ± 0.4 ^cD^	14 ± 2 ^dD^	5.2 ± 0.3 ^aB^
Flavonoids						
Rutin	43.3 ± 0.2 ^dF^	6.3 ± 0.7 ^cB^	5.5 ± 0.1 ^bB^	4.8 ± 0.2 ^abB^	4.7 ± 0.4 ^aB^	4.3 ± 0.3 ^aB^
Epicatechin	19 ± 4 ^D^	n.d.	n.d.	n.d.	n.d.	n.d.
Quercetin 3-glucoside	4.1 ± 0.1 ^bB^	4.2 ± 0.2 ^bA^	3.0 ± 0.2 ^aA^	2.8 ± 0.1 ^aA^	3.0 ± 0.5 ^aA^	n.d.
Quercitrin	traces	traces	traces	traces	traces	3.2 ± 0.8 ^A^
Apigenin-7-glucoside	3.4 ± 0.1 ^aA^	traces	4.7 ± 0.1 ^bB^	7.2 ± 0.3 ^cC^	7.5 ± 0.9 ^cB^	4.7 ± 0.3 ^bB^
Quercetin	3.9 ± 0.1 ^B^	n.d.	n.d.	n.d.	n.d.	n.d.
Naringenin	n.d.	n.d.	6 ± 1 ^bB^	6.5 ± 0.2 ^bC^	7.5 ± 0.9 ^bB^	3.0 ± 0.1 ^aA^
Kaempferol	n.d.	n.d.	n.d.	n.d.	n.d.	n.d.

^a,b,c,d,e^ Different lowercase letters indicate significant differences (*p* < 0.05) between flours. ^A,B,C,D,E,F^ Different capital letters indicate significant differences (*p* < 0.05) between phenolic compounds. n.d.: not detected. Traces: not quantifiable.

## Data Availability

All related data and methods are presented in this paper. Additional inquiries should be addressed to the corresponding author.

## References

[1] Maphosa Y., Jideani V.A. (2017). The Role of Legumes in Human Nutrition. Functional Food-Improve Health through Adequate Food.

[2] Polak R., Phillips E.M., Campbell A. (2015). Legumes: Health Benefits and Culinary Approaches to Increase Intake. Clin. Diabetes.

[3] Stagnari F., Maggio A., Galieni A., Pisante M. (2017). Multiple Benefits of Legumes for Agriculture Sustainability: An Overview. Chem. Biol. Technol. Agric..

[4] Calvo-Lerma J., Asensio-Grau A., García-Hernández J., Heredia A., Andrés A. (2022). Exploring the Impact of Solid-State Fermentation on Macronutrient Profile and Digestibility in Chia (*Salvia hispanica*) and Sesame (*Sesamum indicum*) Seeds. Foods.

[5] Espinosa-Páez E., Alanis-Guzmán M.G., Hernández-Luna C.E., Báez-González J.G., Amaya-Guerra C.A., Andrés-Grau A.M. (2017). Increasing Antioxidant Activity and Protein Digestibility in *Phaseolus vulgaris* and *Avena sativa* by Fermentation with the *Pleurotus ostreatus* Fungus. Molecules.

[6] Hoogeveen G.W.M., Hoogeveen H.W. (2010). System for Solid State Fermentation and Use Thereof.

[7] Lyons M.P., Hoskins B.J. (2014). Compositions and Methods for Conversion of Lignocellulosic Material to Fermentable Sugars and Products Produced Therefrom.

[8] Sánchez-García J., Asensio-Grau A., García-Hernández J., Heredia A., Andrés A. (2022). Nutritional and antioxidant changes in lentils and quinoa through fungal solid-state fermentation with *Pleurotus ostreatus*. Bioresour. Bioprocess..

[9] Abdullah N., Ismail S.M., Aminudin N., Shuib A.S., Lau B.F. (2012). Evaluation of Selected Culinary-Medicinal Mushrooms for Antioxidant and ACE Inhibitory Activities. Evidence-Based Complement. Altern. Med..

[10] Tuck M. (1988). Management of Hypertension in the Patient with Diabetes Mellitus: Focus on the Use of Angiotensin-Converting Enzyme Inhibitors. Am. J. Hypertens..

[11] Marchini M., Carini E., Cataldi N., Boukid F., Blandino M., Ganino T., Vittadini E., Pellegrini N. (2021). The use of red lentil flour in bakery products: How do particle size and substitution level affect rheological properties of wheat bread dough?. LWT-Food Sci. Technol..

[12] Romano A., Gallo V., Ferranti P., Masi P. (2021). Lentil flour: Nutritional and technological properties, in vitro digestibility and perspectives for use in the food industry. Curr. Opin. Food Sci..

[13] Patrón-Vázquez J., Baas-Dzul L., Medina-Torres N., Ayora-Talavera T., Sánchez-Contreras A., García-Cruz U., Pacheco N. (2019). The Effect of Drying Temperature on the Phenolic Content and Functional Behavior of Flours Obtained from Lemon Wastes. Agronomy.

[14] González M., Vernon-Carter E., Alvarez-Ramirez J., Carrera-Tarela Y. (2020). Effects of dry heat treatment temperature on the structure of wheat flour and starch in vitro digestibility of bread. Int. J. Biol. Macromol..

[15] Duan J.-L., Xu J.-G. (2015). Effects of Drying Methods on Physico-Chemical Properties and Antioxidant Activity of Shiitake Mushrooms (*Lentinus edodes*). Agric. Food Sci. Res..

[16] Piskov S., Timchenko L., Grimm W.-D., Rzhepakovsky I., Avanesyan S., Sizonenko M., Kurchenko V. (2020). Effects of Various Drying Methods on Some Physico-Chemical Properties and the Antioxidant Profile and ACE Inhibition Activity of Oyster Mushrooms (*Pleurotus ostreatus*). Foods.

[17] Chkir I., Balti M.A., Ayed L., Azzouz S., Kechaou N., Hamdi M. (2015). Effects of air drying properties on drying kinetics and stability of cactus/brewer’s grains mixture fermented with lactic acid bacteria. Food Bioprod. Process..

[18] Lewis W.K. (1921). The Rate of Drying of Solid Materials. J. Ind. Eng. Chem..

[19] Association of Official Analysis Chemists (2000). AOAC Official Methods of Analysis of AOAC International.

[20] Miller G.L. (1959). Use of Dinitrosalicylic Acid Reagent for Determination of Reducing Sugar. Anal. Chem..

[21] Sansano M., Juan-Borrás M., Escriche I., Andrés A.M., Heredia A. (2015). Effect of Pretreatments and Air-Frying, a Novel Technology, on Acrylamide Generation in Fried Potatoes. J. Food Sci..

[22] Aidoo K.E., Hendry R., Wood B.J.B. (1981). Estimation of fungal growth in a solid state fermentation system. Eur. J. Appl. Microbiol. Biotechnol..

[23] Scotti C., Vergoignan C., Feron G., Durand A. (2001). Glucosamine measurement as indirect method for biomass estimation of *Cunninghamella elegans* grown in solid state cultivation conditions. Biochem. Eng. J..

[24] Haug W., Lantzsch H.-J. (1983). Sensitive method for the rapid determination of phytate in cereals and cereal products. J. Sci. Food Agric..

[25] Wu P., Zhao T., Tian J.-C. (2010). Phytic Acid Contents of Wheat Flours from Different Mill Streams. Agric. Sci. China.

[26] Caprioli G., Nzekoue A.F.K., Giusti F., Vittori S., Sagratini G. (2018). Optimization of an extraction method for the simultaneous quantification of sixteen polyphenols in thirty-one pulse samples by using HPLC-MS/MS dynamic-MRM triple quadrupole. Food Chem..

[27] Giusti F., Capuano E., Sagratini G., Pellegrini N. (2019). A comprehensive investigation of the behaviour of phenolic compounds in legumes during domestic cooking and in vitro digestion. Food Chem..

[28] Tanleque-Alberto F., Juan-Borrás M., Escriche I. (2019). Antioxidant characteristics of honey from Mozambique based on specific flavonoids and phenolic acid compounds. J. Food Compos. Anal..

[29] Chang C.-H., Lin H.-Y., Chang C.-Y., Liu Y.-C. (2006). Comparisons on the antioxidant properties of fresh, freeze-dried and hot-air-dried tomatoes. J. Food Eng..

[30] Thaipong K., Boonprakob U., Crosby K., Cisneros-Zevallos L., Hawkins Byrne D. (2006). Comparison of ABTS, DPPH, FRAP, and ORAC assays for estimating antioxidant activity from guava fruit extracts. J. Food Compos. Anal..

[31] Sharma S., Kataria A., Singh B. (2022). Effect of thermal processing on the bioactive compounds, antioxidative, antinutritional and functional characteristics of quinoa (*Chenopodium quinoa*). LWT.

[32] Akıllıoğlu H.G., Karakaya S. (2009). Effects of heat treatment and in vitro digestion on the Angiotensin converting enzyme inhibitory activity of some legume species. Eur. Food Res. Technol..

[33] Hernández-Olivas E., Muñoz-Pina S., García-Hernández J., Andrés A., Heredia A. (2022). Impact of common gastrointestinal disorders in elderly on in vitro meat protein digestibility and related properties. Food Biosci..

[34] Aguilera Y., Dueñas M., Estrella I., Hernández T., Benitez V., Esteban R.M., Martín-Cabrejas M.A. (2010). Evaluation of Phenolic Profile and Antioxidant Properties of Pardina Lentil as Affected by Industrial Dehydration. J. Agric. Food Chem..

[35] Chawla P., Bhandari L., Sadh P.K., Kaushik R. (2017). Impact of Solid-State Fermentation (*Aspergillus oryzae*) on Functional Properties and Mineral Bioavailability of Black-Eyed Pea (*Vigna unguiculata*) Seed Flour. Cereal Chem..

[36] Mora-Uzeta C., Cuevas-Rodríguez E., López-Cervantes J., Milán-Carrillo J., Gutiérrez-Dorado R., Reyes-Moreno C. (2019). Improvement Nutritional/Antioxidant Properties of Underutilized Legume Tepary Bean (*Phaseolus acutifolius*) by Solid State Fermentation. Agrociencia.

[37] Garrido-Galand S., Asensio-Grau A., Calvo-Lerma J., Heredia A., Andrés A. (2021). The potential of fermentation on nutritional and technological improvement of cereal and legume flours: A review. Food Res. Int..

[38] Plaza J., Morales-Corts M., Pérez-Sánchez R., Revilla I., Vivar-Quintana A. (2021). Morphometric and Nutritional Characterization of the Main Spanish Lentil Cultivars. Agriculture.

[39] Guiné R.P.F. (2018). The Drying of Foods and Its Effect on the Physical-Chemical, Sensorial and Nutritional Properties. ETP Int. J. Food Eng..

[40] Choe U., Osorno J.M., Ohm J.-B., Chen B., Rao J. (2022). Modification of physicochemical, functional properties, and digestibility of macronutrients in common bean (*Phaseolus vulgaris* L.) flours by different thermally treated whole seeds. Food Chem..

[41] Yi J.-Y., Lyu J., Bi J.-F., Zhou L.-Y., Zhou M. (2017). Hot air drying and freeze drying pre-treatments coupled to explosion puffing drying in terms of quality attributes of mango, pitaya, and papaya fruit chips. J. Food Process. Preserv..

[42] Djekic I., Tomic N., Bourdoux S., Spilimbergo S., Smigic N., Udovicki B., Hofland G., Devlieghere F., Rajkovic A. (2018). Comparison of three types of drying (supercritical CO_2_, air and freeze) on the quality of dried apple—Quality index approach. LWT.

[43] Nowak D., Jakubczyk E. (2020). The Freeze-Drying of Foods—The Characteristic of the Process Course and the Effect of Its Parameters on the Physical Properties of Food Materials. Foods.

[44] Ranilla L.G., Genovese M.I., Lajolo F.M. (2009). Effect of Different Cooking Conditions on Phenolic Compounds and Antioxidant Capacity of Some Selected Brazilian Bean (*Phaseolus vulgaris* L.) Cultivars. J. Agric. Food Chem..

[45] Madhujith T., Shahidi F. (2005). Beans: A Source of Natural Antioxidants. Phenolic Compounds in Foods and Natural Health Products.

[46] Gan R.-Y., Lui W.-Y., Chan C.-L., Corke H. (2016). Hot Air Drying Induces Browning and Enhances Phenolic Content and Antioxidant Capacity in Mung Bean (*Vigna radiata* L.) Sprouts. J. Food Process. Preserv..

[47] Anton A.A., Ross K.A., Beta T., Fulcher R.G., Arntfield S.D. (2008). Effect of pre-dehulling treatments on some nutritional and physical properties of navy and pinto beans (*Phaseolus vulgaris* L.). LWT.

[48] Zou Y., Gao Y., He H., Yang T. (2018). Effect of roasting on physico-chemical properties, antioxidant capacity, and oxidative stability of wheat germ oil. LWT.

[49] Que F., Mao L., Fang X., Wu T. (2008). Comparison of hot air-drying and freeze-drying on the physicochemical properties and antioxidant activities of pumpkin (*Cucurbita moschata* Duch.) flours. Int. J. Food Sci. Technol..

[50] Bajpai B., Patil S. (2008). A New Approach to Microbial Production of Gallic Acid. Braz. J. Microbiol..

[51] Aguilar-Zárate P., Cruz-Hernández M., Montañez J., Belmares-Cerda R., Aguilar C. (2014). Bacterial tannases: Production, properties and applications, Tanasas bacterianas: Producción, propiedades y aplicaciones. Rev. Mex. Ing. Química.

[52] Thavarajah P., Thavarajah D., Vandenberg A. (2009). Low Phytic Acid Lentils (*Lens culinaris* L.): A Potential Solution for Increased Micronutrient Bioavailability. J. Agric. Food Chem..

[53] Farinde E.O., Olanipekun O.T., Olasupo R.B. (2018). Nutritional Composition and Antinutrients Content of Raw and Processed Lima Bean (*Phaseolus lunatus*). Ann. Food Sci. Technol..

[54] Barahuie F., Dorniani D., Saifullah B., Gothai S., Hussein M.Z., Pandurangan A.K., Arulselvan P. (2017). Sustained release of anticancer agent phytic acid from its chitosan-coated magnetic nanoparticles for drug-delivery system. Int. J. Nanomed..

[55] Shamsuddin A.M. (2002). Anti-cancer function of phytic acid. Int. J. Food Sci. Technol..

[56] Schröterová L., Hašková P., Rudolf E., Cervinka M. (2010). Effect of phytic acid and inositol on the proliferation and apoptosis of cells derived from colorectal carcinoma. Oncol. Rep..

[57] Gu M., Roy S., Raina K., Agarwal C., Agarwal R. (2009). Inositol Hexaphosphate Suppresses Growth and Induces Apoptosis in Prostate Carcinoma Cells in Culture and Nude Mouse Xenograft: PI3K-Akt Pathway as Potential Target. Cancer Res..

[58] Vucenik I., Shamsuddin A.M. (2006). Protection Against Cancer by Dietary IP_6_and Inositol. Nutr. Cancer.

[59] Hong F., Ming L., Yi S., Zhanxia L., Yongquan W., Chi L. (2008). The Antihypertensive Effect of Peptides: A Novel Al-ternative to Drugs?. Peptides.

[60] Ansor N.M., Abdullah N., Aminudin N. (2013). Anti-angiotensin converting enzyme (ACE) proteins from mycelia of *Ganoderma lucidum* (Curtis) P. Karst. BMC Complement. Altern. Med..

[61] Lin Z., Lai J., He P., Pan L., Zhang Y., Zhang M., Wu H. (2023). Screening, ACE-inhibitory mechanism and structure-activity relationship of a novel ACE-inhibitory peptide from *Lepidium meyenii* (Maca) protein hydrolysate. Food Biosci..

